# Cnidarian toxins: omics approaches and recombinant proteins

**DOI:** 10.1590/1678-9199-JVATITD-2025-0019

**Published:** 2025-12-19

**Authors:** Jesús Eduardo Vega-Tamayo, Esteban de Jesús Alcantar-Orozco, Ramiro Arturo Mendoza-Ramírez, Luna Gabriela Silva-Díaz, Jorge Alberto Aguilar-Salazar, Diego Hermilo Salvador-Aguilar, Alejandra Rojas-Molina

**Affiliations:** 1 School of Chemistry, Autonomous University of Querétaro, Cerro de las Campanas, Querétaro, Qro, Mexico.; 2 Laboratory of Chemical and Pharmacological Research of Natural Products, School of Chemistry, Autonomous University of Querétaro, Cerro de las Campanas, Querétaro, Qro, Mexico.

**Keywords:** Cnidarians, Toxins, Omics sciences, Recombinant proteins

## Abstract

Cnidarian venom toxins have attracted increasing interest due to their remarkable molecular diversity and pharmacological potential. Omics technologies - such as genomics, transcriptomics, proteomics, and metabolomics - have facilitated the identification of toxin-encoding genes, providing key insights into their evolutionary trajectories and structure-function relationships, which are essential for understanding their mechanisms of action and therapeutic value. Nevertheless, the functional validation and production of complex toxins remain challenging, particularly for those requiring intricate folding or post-translational modifications. Recombinant expression has emerged as a strategic alternative to traditional purification methods, enabling controlled toxin production and the possibility of modifying their properties through genetic engineering. In parallel, advances in synthetic biology, such as cell-free protein synthesis systems, are creating new opportunities for toxin characterization, although their industrial scalability remains limited. Computational tools, including those based on artificial intelligence, are beginning to support the prioritization and functional analysis of toxins identified through omics approaches. This review provides an updated overview of the advances, limitations, and future perspectives in cnidarian toxin research, highlighting their promising role as a valuable source of bioactive compounds with therapeutic and biotechnological applications.

## Background

**Figure 1.**
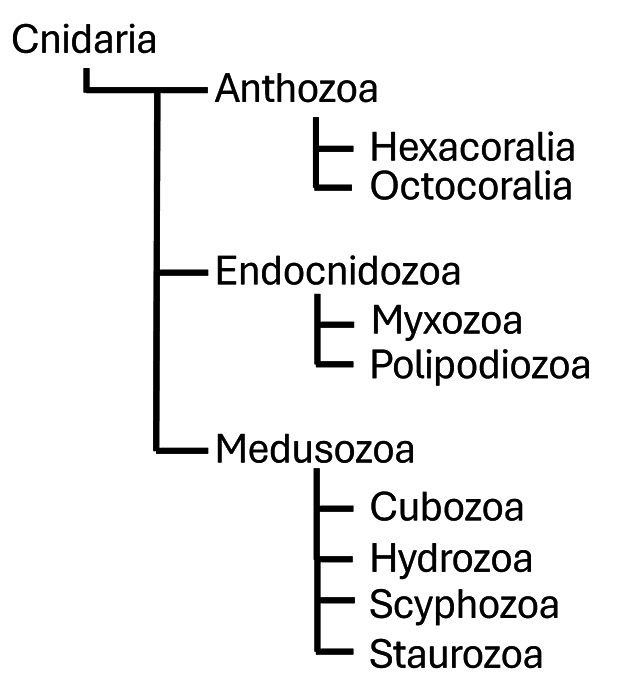
Phylogenetic relationships within the phylum Cnidaria, including the subphyla Anthozoa, Endocnidozoa, and Medusozoa, and their respective classes. Diagram elaborated based on the taxonomic classification provided by the World Register of Marine Species (WoRMS) [3].

The phylum Cnidaria comprises a diverse group of venomous aquatic animals that have existed for approximately one billion years and includes around 10,000 species distributed worldwide in shallow marine environments. This phylum is divided into three main subphyla, each characterized by distinct morphological traits and life forms (Figure 1). Anthozoa includes corals and sea anemones, which are known for their remarkable diversity of shapes and colors and play a crucial role in the formation of marine ecosystems. Medusozoa comprises organisms such as jellyfish, distinguished by their gelatinous bodies, long tentacles, and ability to swim freely. Lastly, Endocnidozoa is a recently identified group that includes highly simplified parasitic species (e.g., Myxozoa), which inhabit their hosts and have lost many of the typical features of cnidarians [1,2].

These three subphyla underscore the remarkable diversity of cnidarians, including forms that range from sessile organisms to active swimmers, and from habitat builders to highly specialized parasites. Despite their diversity, all these organisms share a common structure: the polyp. This structural organization consists of a gastrovascular cavity surrounded by three layers. The internal endodermis is responsible for nutrient absorption and waste excretion, while the external ectodermis mediates environmental interactions. Separating these two cellular layers is the mesoglea, a fibrillar matrix that provides structural support [4-6].

Cnidarians also exhibit two distinct life cycle patterns, depending on the subphylum. In Anthozoa, the life cycle comprises three stages: embryo, larva, and polyp (the sessile form). In contrast, the life cycle of Medusozoa consists of four phases: embryo, larva, polyp, and medusa (the free-swimming form) [7]. Regarding nutrition, although most cnidarians are predators, some species acquire their nutrients from carrion or from symbiotic associations with algae of the Symbiodiniaceae family [8]. However, one of the most distinctive evolutionary features of this phylum is the presence of stinging cells known as cnidocytes, which contain a specialized organelle called cnidocyst (or cnid), responsible for the production, storage and delivery of venom [9,10].

## Stinging cells

Cnidocytes exhibit variations in both morphology and specialized functions, including prey capture and immobilization, as well as the deterrence and repulsion of predators and competitors [11,12]. These cells contain cnidae or cnidocysts, which develop through the growth of a vesicle formed by the aggregation of protein-containing vesicles derived from the Golgi apparatus. The tubules of cnidocysts originate through membrane tubulation at the apical site of the vesicle. Once this process is complete, the tubule invaginates into the capsule matrix, which is sealed by a lid-like structure known as the operculum. The tubule spines develop after invagination, and final cnidocyst maturation occurs when the capsule wall undergoes compaction through the polymerization of structural proteins such as minicollagens [13,14].

Cnidocysts consist of a collagen-walled capsule that stores venom, a cnidocil functioning as a mechanoreceptor, an operculum, and a hollow, coiled tubule responsible for discharging the capsule’s contents. These organelles are classified into three main types based on their axial structure, as well as the type and distribution of their spines: nematocysts, spirocysts, and ptychocysts [5,15]. Nematocysts, present in all cnidarians, are specialized for venom injection into target organisms and represent the most morphologically and functionally diverse group of cnidocysts [16].

In contrast, spirocysts, found primarily in members of the class Anthozoa, do not contain toxins. The spirocyst capsule has a thin wall, and its tubule is helically coiled. Although it lacks spines, the tubule contains adherent hydroscopic substances that mechanically immobilize the prey, facilitating capture. Lastly, ptychocysts, characteristic of the Anthozoa class, also lack spines and function as adhesive structures for prey attachment [17,18].

**Figure 2.**
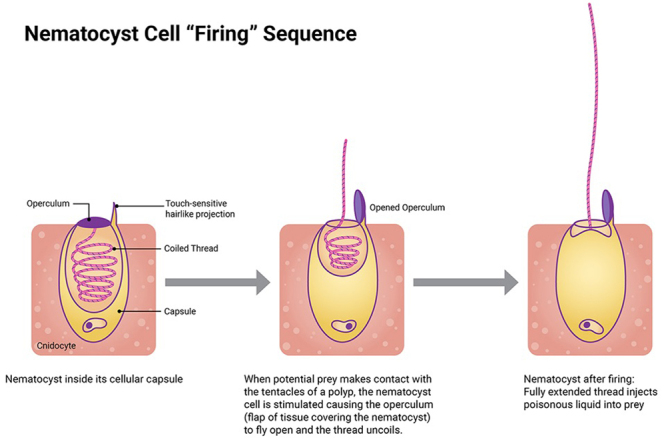
Illustration showing the structure and discharge sequence of a nematocyst. Image courtesy of the NOAA Nartional Ocean Service [20].

The nematocyst discharge mechanism is triggered by external mechanical or chemical stimuli, leading to a temporary rise in intracapsular osmotic pressure. This occurs due to cnidocyte exposure to an external solution, followed by the exocytosis of cations from the capsule. These osmotic pressure differences across the capsule wall are maintained until the intracapsular pressure exceeds a critical threshold, triggering nematocyst discharge. During this process, the tubule’s inner surface inverts, exposing its venom-containing lumen. This allows the interior of the tubule to remain continuous with the interior of the capsule, thereby facilitating venom expulsion (Figure 2) [12,17,19].

## Venom composition

Cnidarians are known for their potent venom, which has been extensively studied through analytical and clinical research, revealing remarkable a remarkable diversity of toxicological properties [12,21]. This toxicological arsenal ranges from biogenic amines like serotonin and histamine to peptide neurotoxins that modulate ion channels and high molecular mass proteins with enzymatic or pore-forming activity [22,23]. Several of these toxins are produced as pre-toxins, and some share structural and functional similarities with those found in other venomous animals, such as Kunitz-like peptides and Kv1 potassium channel blockers, which show convergent evolution in scorpions, sea anemones, and other organisms. the use of omics and biotechnological tools to study their toxins, highlighting their health and pharmaceutical implications. Table 1 presents the main toxins identified in cnidarian venoms between 2019 and 2024 using omics approaches.

**Table 1.** Cnidarian toxins identified from 2019 to 2024.

Toxin	Organisms	Reference
**Phospholipases A2**		
A2-PLTX-Pcb1a	*Palythoa carbaeorum*	[24]
Dermonecrotic toxin StSicToxbetaIF1 (Fragment)	*Nemopilema nomurai*	[25]
Basic phospholipase A2 homolog	*N. nomurai*	[25]
PLA2 (Conodipine-M) (n =3)	*Buddenbrockia plumatellae*	[26]
Plancitoxin-1 (n = 3)	*B. plumatellae*	[26]
Phospholipase A2 A2-actitoxin-Cgg2a	*Polypodium hydriforme*	[26]
Phospholipase A2	*Cyanea* sp.	[27]
85/88 kDa calcium-independent phospholipase A2	*Cyanea* sp.	[27]
Phospholipases A2 (n = 9)	*Rhopilema esculentum*	[28]
Acidic phospholipase A2 PA4 (n = 4)	*R. esculentum*	[28]
Phospholipase A2 isozymes PA3A/PA3B/PA5	*R. esculentum*	[28]
Putative phospholipase B-like 2	*R. esculentum*	[28]
Phospholipase A2 A2-actitoxin-Ucs2a	*Actinia equina*	[29]
Phospholipase A2	*N. nomurai*	[30]
Phospholipase D gamma 2	*N. nomurai*	[30]
Phospholipases A2 (n = 11)	*Galaxea fascicularis*	[31]
Phospholipases A2 (n = 2)	*Olindias sambaquiensis*	[18]
Phospholipases A2 (n = 23)	*Phacellophora camtschatica*	[32]
Phospholipases A2 (n = 3)	*Ectopleura larynx*	[33]
Phospholipase A2	*Calliactis polypus*	[34]
Phospholipases A2 (n = 12)	*N. nomurai*	[35]
Phospholipases A2 (n = 2)	*Hydractinia symbiolongicarpus*	[36]
**Metallopeptidases**		
JVMP17-1	*N. nomurai*	[37]
JVMP17-2	*N. nomurai*	[37]
Metallopeptidases (n = 90)	*P. hydriforme*	[26]
Metallopeptidases (n = 43)	*B. plumatellae*	[26]
Matrix metalloproteinase (n = 2)	*Cyanea* sp.	[27]
Zinc metalloproteinase nas-14	*Cyanea* sp.	[27]
ADMTM (n = 2)	*Cyanea* sp.	[27]
Snake venom metalloproteinase-disintegrin-like mocarhagin	*Cyanea* sp.	[27]
Zinc metalloproteinases nas (n = 39)	*R. esculentum*	[28]
Disintegrin and metalloproteinase (n = 21)	*R. esculentum*	[28]
Metallopeptidases (n = 15)	*A. equina*	[29]
ATP-dependentzinc metallopeptidaseYME1homolog	*N. nomurai*	[30]
Snake venom metalloproteinase	*N. nomurai*	[30]
A disintegrin and metallo-proteinase with thrombo-spondin motifs (n = 4)	*N. nomurai*	[30]
ATP-dependentzinc metallopeptidase FtsH	*N. nomurai*	[30]
Disintegrin and metalloproteinase domain-containing protein17	*N. nomurai*	[30]
Zinc metalloproteinase nas-8	*N. nomurai*	[30]
Zinc metalloproteinase-disintegrin-like halysase	*G. fascicularis*	[31]
Astacin-like metallopeptidase toxin	*G. fascicularis*	[31]
Metallopeptidases (n = 83)	*Exaiptasia diaphana*	[38]
Snake venom metalloproteinase ACLF	*O. sambaquiensis*	[18]
Metalloproteinases (n = 31)	*Phacellophora camtschatica*	[32]
Metalloproteinases (n = 35)	*E. larynx*	[33,35]
Reprolysin (M12B) family zinc metallopeptidases (n = 23)	*N. nomurai*	[35]
Metalloproteinases (n = 12)	*H. symbiolongicarpus*	[36]
**Other enzymatic toxic components**		
Phospholipases A1 (n = 4)	*P. hydriforme*	[26]
Phospholipases B (n = 3)	*B. plumatellae*	[26]
Phospholipase B	*Ceriantharia* spp*.*	[39]
Phospholipase B	*Millepora alcicornis*	[40]
Phospholipase B	*H. symbiolongicarpus*	[36]
Phospholipases B (n = 3)	*G. fascicularis*	[31]
Phospholipases B (n = 3)	*E. diaphana*	[38]
Phospholipases B (n = 2)	*N. nomurai*	[35]
Putative phospholipase B-like 2	*R. esculentum*	[28]
Phospholipase B1	*Cyanea* sp.	[27]
Phospholipase D	*G. fascicularis*	[31]
Phospholipases D (n = 4)	*O. sambaquiensis*	[18]
Phospholipase D1	*Cyanea* sp.	[27]
Serine-peptidase	*Millepora complanate*	[41]
Serine-peptidases (n = 11)	*B. plumatellae*	[26]
Serine-peptidases (n = 18)	*P. hydriforme*	[26]
Serine-peptidases (n = 2)	*Cyanea* sp.	[27]
Serine-peptidases (n = 7)	*R. esculentum*	[28]
Serine-peptidases (n = 3)	*A. equina*	[29]
Snake venom serine peptidases Nikobin	*N. nomurai*	[30]
Serine-peptidase	*E. diaphana*	[38]
Snake venom serine peptidase 1	*O. sambaquiensis*	[18]
Venom serine carboxypeptidase	*O sambaquiensis*	[18]
Serine-peptidases (n = 22)	*P. camtschatica*	[32]
Serine-peptidases (n = 23)	*E. larynx*	[33]
**Neurotoxins**		
RTX-VI	*Heteractis crispa*	[42]
Kappa-stichotoxin-Shd1a/kappastichotoxin-Shd1b	*N. nomurai*	[25]
Type III potassium channel toxin protein	*A. equina*	[29]
Potassium channel toxin BmTXK-beta	*A. equina*	[29]
Potassium channel toxin alpha-KTx 6.2	*A. equina*	[29]
Ly-6	*Velella velella*	[43]
Alfa-elapitoxina-Dpp2a	*V. velella*	[43]
Kappa-esticotoxina-She3a	*V. velella*	[43]
Potassium channel toxins alpha-Ktx (n = 2)	*O. sambaquiensis*	[18]
Putative potassium channel blocker TXKS1	*O. sambaquiensis*	[18]
Ion channel impairing toxins (n = 26)	*E. larynx*	[33]
NaTx type I	*C. polypus*	[34]
KTx type I	*C. polypus*	[34]
Hau-IV	*Heteractis aurora*	[44]
**Kunitz-type inhibitors**		
HCIQ1c9	*H. crispa*	[45]
HCIQ2c1	*H. crispa*	[45]
HCIQ4c7	*H. crispa*	[45]
HMIQ3c1	*Heteractis magnifica*	[45]
Kunitz-type serine peptidase inhibitor 5	*P. hydriforme*	[26]
Kunitz-type serine peptidase inhibitor kunitoxin-Phi1 (n = 2)	*P. hydriforme*	[26]
Serine peptidase inhibitor (kunitz-like) (n = 23)	*B. plumatellae*	[26]
AsKC11	*Anemonia sulcata*	[46]
Kunitz-type serine peptidase inhibitor mulgin-2	*Cyanea* sp.	[27]
Kunitz-type serine peptidase inhibitor	*R. esculentum*	[28]
Kunitz-type serine peptidase inhibitor long epsilon-dendrotoxin Arg55	*A. equina*	[29]
Putative Kunitz-type serine peptidase inhibitor	*N. nomurai*	[30]
Kunitz-type kappaPI-theraphotoxin	*G. fascicularis*	[31]
Kunitz-type (n = 10)	*E. diaphana*	[38]
Kunitz-type serine peptidase inhibitor bitisilin-3	*O. sambaquiensis*	[18]
Kunitz-type serine peptidase inhibitor	*E. larynx*	[33]
Kunitz/Bovine pancreatic trypsin inhibitor domain (n = 5)	*N. nomurai*	[35]
**TRPV1 channel inhibitors**		
PcActx	*P. caribaeorum*	[47]
Tst2	*Telmatactis stephensoni*	[48]
**TRPA1 channel modulators**		
Hau-II & Hau-III	*H. aurora*	[44]
**ASIC channel modulators**		
Hmg 1b-2, Hmg 1b-4 & Hmg 1b-5	*H. magnifica*	[49,50]
**Small Cysteine-Rich Proteins**		
Hact_SCRiP1	*Heliofungia actiniformis*	[51]
Hact-4	*H. actiniformis*	[51]
**Pore-forming toxins**		
Alciporin	*M. alcicornis*	[52]
XaxB	*V. velella*	[43]
Conoporin-Cn1	*O. sambaquiensis*	[18]
U7-lycotoxin-Ls1e	*O. sambaquiensis*	[18]
PFTs (n = 5)	*E. larynx*	[33]
**Others**		
Sco 9a-1	*Stomphia coccinea*	[53]
Ms11a-1 - Ms11a-4	*Metridium senile*	[54]
Hau-I	*H. aurora*	[44]

The toxins classified under “Others” have not yet been fully characterized and therefore cannot be assigned to any of the eight categories established in this or other publications. The number in parenthesis indicates the number of toxins of same type identified in the study.

## Enzymes with toxic activity in cnidarian venoms

Enzymes with toxic activity constitute a key component of cnidarian venoms, playing crucial roles in tissue degradation, induction of hemolysis, dissemination of other toxins, and modulation of the host response. Among the most represented enzymatic families are phospholipases A2 (PLA2s), metallopeptidases, and other enzymes such as serine peptidases, collagenases, elastases, hyaluronidases, and phospholipases B. 

Phospholipases A2 are widely distributed across animals, plants, fungi, and bacteria. In cnidarians, they have been associated with functions such as digestion, defense, and hemolytic activity. Within this enzyme superfamily, two main groups are recognized: cytosolic PLA2 (cPLA2s) and secreted PLA2 (sPLA2s). The latter typically range from 13 to 19 kDa in molecular mass and are responsible for hydrolyzing the sn-2 acyl bond of glycerophospholipids. sPLA2s, which are found across various lineages of venomous animals, have been implicated in inflammatory, neurotoxic, and tumorigenic processes, suggesting the convergent recruitment of endogenous proteins into venoms [24-29]. Multiple toxic PLAs have been described in cnidarians from the Anthozoa, Scyphozoa, Hydrozoa, and Cubozoa subphyla. For instance, Becerra-Amezcua et al. [55] demonstrated that hemolysis induced by the venom of jellyfish form the genus *Chrysaora* was mediated by PLA2, while Cuevas-Cruz et al. [24] isolated an sPLA2 with neurotoxic activity from the sea anemone *P. caribaeorum*. PLA2s have also been identified through transcriptomic and proteomic analyses in *P. hydriforme*, *B. plumatellae*, and *Cyanea* sp. [32,33], and genomic analysis of *R. esculentum* revealed 18 PLA2-coding genes [34]. In *A. equina*, PLA2s have been reported in specific morphotypes as well [35].

Metallopeptidases represent another important class of toxic enzymes that contribute to hemorrhage, necrosis, and toxin dissemination by degrading components of the extracellular matrix and interfering with blood coagulation [36]. In jellyfish, these enzymes have shown proteolytic activity against substrates such as gelatin, casein and fibrinogen [22]. In *Nematostella vectensis*, a zinc-dependent astacin-like metallopeptidase has been reported to be expressed in cnidocytes [37], while a proteomic study of *M. complanata* identified a metallopeptidase homologous to astacins, a family known to induce endothelial cell death and to degrade fibrinogen, fibronectin, and gelatin [36]. Moreover, two metallopeptidase isoforms (JVMP17-1 and JVPM17-2) from the venom of *N. nomurai* exhibited dermotoxic and cytotoxic effects [37]. Numerous metallopeptidases have been identified at the transcriptomic and proteomic levels in cnidarians such as *P. hydriforme*, *B. plumatellae*, *Cyanea* sp., and *R. esculentum*, with up to 60 genes associated with this enzymatic family detected in the latter [32-34].

In addition to PLA2s and metallopeptidases, cnidarian venoms contain other enzymes with toxic activity which although less abundant play significant roles in venom pathogenicity. Phospholipase B (PLB), for example, contributes to the degradation of cellular membranes and the induction of necrosis and inflammation. PLBs have been reported in *Physalia physalis* and *M. alcicornis* [39-41]. Serine peptidases, which trigger inflammation, disrupt coagulation, and degrade structural proteins, have been detected in *Rhopilema nomadica*, *M. complanata*, *Cyanea* sp., and *A. equina* [32-35,43,44].

Collagenases, elastases, and hyaluronidases have also been identified in cnidarian venoms. These enzymes degrade collagen, elastin, and hyaluronic acid, respectively, facilitating toxin penetration and diffusion. They are present in species such as *P. physalis*, *Chrysaora quinquecirrha*, *Chironex fleckeri*, *Cyanea capillata*, *N. nomurai*, *Cyanea nozakii*, and *Aurelia aurita* [5,22,45]. Other potentially toxic enzymes, including lipases, hydrolases, and oxidases, have been identified in *P. hydriforme* and *B. plumatellae*, with a higher representation in the former [32]. The genome of *R. esculentum* also revealed genes encoding nucleotidases, dipeptydil peptidases, and prothrombin-like proteins [28,56]. 

## Neurotoxins targeting voltage-gated Na⁺ and K⁺ channels

Ion channels encompass multiple subtypes with distinct physiological, pharmacological, and structural properties. Among them, voltage-gated ion channels play a crucial role in cell excitability and neuromuscular signal transmission by regulating ion flow through membrane pores via an electrochemical gradient. Disruptions in this mechanism can significantly alter signal transmission to neurons and muscles [57,58]. Neurotoxins, for instance, directly affect ion channels and receptors, neuromuscular junctions and nerve cell membranes, leading to paralysis [59]. In fact, there is a wide variety of venomous animals that have developed neurotoxins that can interact with ion channels to immobilize their prey. Within cnidarians such as sea anemones, these toxins are among the most well-characterized venom components [60]. In this context, toxins targeting voltage-gated K⁺ and Na⁺ channels (Kv and Nav, respectively) constitute one of the most diverse and functionally significant groups of neurotoxins [61].

Neurotoxins that interact with Nav channels (NaTx) have a molecular mass ranging from 3.5 to 6.5 kDa and bind to site 3 of the Nav channel to regulate its function. By controlling channel opening and closing, these toxins modulate electrical signaling [62-64]. NaTx toxins are classified into four groups based on their binding site on the channel. Type I NaTx includes AeI and AETX-I from *A. equina* and *Anemia eritrea*, while type II NaTx comprises RTX (I-V) from *H. crispa* and Rp (II-III) from *Radianthus paumotensis*. Type III NaTx includes Av3 from *Anemonia viridis* and Er-I from *Entacmaea ramsayi*, whereas type IV NaTx consists of isotoxins CLX-I and CLX-II from the small anemone *Calliactis parasitica* [65].

**Figure 3.**
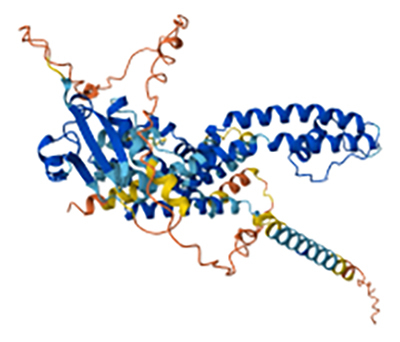
Predicted 3D-structure of a toxin from the sea urchin *Strongylocentrotus purpuratus* that interacts with a voltage-gated potassium channel (UniProt ID: Q8I4B0). The structure was generated using AlphaFold v2.0 and visualized with Mol* Viewer. High-confidence regions are shown in blue, whereas lower-confidence regions are depicted in red. Image courtesy of Jumper et al. [72] and Varadi et al. [73].

On the other hand, toxins that interact with Kv channels (KTxs) have a molecular mass between 3 and 6 kDa. These toxins act either by directly blocking the channel or by modifying its activation kinetics, leading to electrical signal dysfunction due to sustained depolarization (Figure 3) [58,66]. KTxs are classified into five types. Type I KTxs target Kv1 and Kv3 channels, including the Bg toxin from *Bunodosoma granulifera* and AeK from *A. equina* [64,67]. Type II KTxs act on Kv1.2 channels, such as kalicludin 1-3 from *A. sulcata* and HmK from *Radianthus magnifica* [64,68]. Type III KTxs affect Kv3.4 channels, with BDS I and II from *A. viridis* [64]. Type IV KTxs include Bcg-III-23.41 from *Bunodosoma cangicum* and SHTX I-III from *Stichodactyla haddoni* [12,69,70], while type V KTxs comprise the BcsTx3 toxin from *Bunodosoma caissarum* [12,71].

## Kunitz-type inhibitors

**Figure 4.**
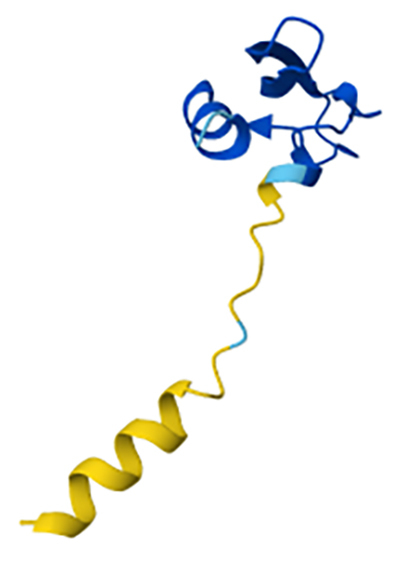
Predicted 3D-structure of PI-stichotoxin-She3a, a Kunitz-type serine peptidase inhibitor from the sea anemone *Stichodactyla helianthus*, generated using AlphaFold v2.0. The structure reveals a conserved Kunitz fold stabilized by disulfide bonds. The model is available in the AlphaFold Proteins Structure Database (AF-P0DMJ5-F1). Image courtesy of Jumper et al. [72] and Varadi et al.[73].

Kunitz-type peptidase inhibitors (KTPIs) are peptides widely distributed among venomous organisms, both terrestrial and marine, playing a crucial role in peptidase regulation and ion channel modulation. These peptides, typically composed of 50-60 amino acids, exhibit a compact structure characterized by the Kunitz motif, which includes three highly conserved disulfide bonds: CysI-CysVI, CysII-CysIV, and CysIII-CysV. Their mechanism of action involves competitive binding to the active site of serine peptidases, leading to direct inhibition (Figure 4) [45,74].

In the phylum Cnidaria, particularly in sea anemones, toxins containing domains homologous to Kunitz-type inhibitors have been identified. These molecules not only inhibit serine peptidases but also block voltage-gated potassium (Kv) channels, thereby contributing to both prey immobilization and defense against predators. A representative example is the kalicludins, peptides isolated from sea anemones that exhibit this dual functionality by inhibiting peptidases and blocking Kv1.2 channels [68]. Moreover, it has been suggested that these inhibitors may protect toxins from rapid degradation within the prey’s body, thereby enhancing their effectiveness during envenomation. 

The study of these inhibitors in cnidarians has revealed remarkable functional diversity. For instance, in *H. crispa*, peptides such as InhVJ, HCRG1, and HCRG2 have been identified, with the latter two capable of blocking voltage-gated potassium channels [75]. Recent studies on *H. crispa* and *H. magnifica* have described four Kunitz-type peptides (HCIQ1c9, HCIQ2c1, HCIQ4c7, and HMIQ3c1) exhibiting neuroprotective activity [45]. The identification of these peptides underscores the sophistication of predation and defense mechanisms in cnidarians, highlighting the significance of Kunitz inhibitors in their biology and ecology.

Kunitz-type inhibitors found in cnidarians have been studied for their therapeutic potential. A notable example is the protein ShPI-1, isolated from the sea anemone *S. helianthus*. This protein exhibits blocking activity on voltage-gated potassium channels, specifically Kv1.1, Kv1.2, and Kv1.6. Its dual functionality suggests potential applications in treating conditions where peptidase regulation and ion channel modulation are relevant, such as inflammatory processes and neurological disorders. The structure of ShPI-1 closely resembles that of bovine pancreatic trypsin inhibitor (BPTI), the prototypical Kunitz-type inhibitor. This structural similarity is key to its ability to inhibit peptidases and block potassium channels. Structure-function relationship studies have shown that modifications in specific regions of the molecule can alter its affinity for peptidases and ion channels, opening possibilities for designing variants with enhanced therapeutic properties [76].

## TRPV1 channel inhibitors and TRPA1 channel modulators

TRPV1 (Transient Receptor Potential Vanilloid 1) and TRPA1 (Transient Receptor Potential Ankyrin 1) channels belong to the TRP (Transient Receptor Potential) ion channel family, which responds to various noxious stimuli, including temperature changes, pH variations, and irritant compounds. These channels play a crucial role in nociception and inflammation and are overexpressed in pathological conditions such as chronic inflammation and neuropathic pain [77,78]. TRPV1 is activated by capsaicin, heat, and acidosis, while TRPA1 responds to a broad range of chemical irritants [79].

Recent studies have identified bioactive compounds derived from marine organisms, including cnidarians, that inhibit or modulate the activity of these ion channels. One example is the peptide HCRG21, extracted from the venom of the jellyfish *R. esculentum*, which has been shown to be a potent TRPV1 channel modulator. HCRG21 significantly reduces the inflammatory response by inhibiting capsaicin-induced channel activation [80].

Regarding TRPA1 modulators, peptides that influence the activity of this channel have been identified in sea anemones. For instance, the peptide BDS-I, isolated from *A. sulcata*, has been shown to inhibit both TRPV1 and TRPA1. Studies in animal models have demonstrated that BDS-I reduces neuronal excitability and alleviates pain perception in response to chemical irritants [81]. Another peptide of interest is Hcr 1b-1, derived from *H. crispa*. Although initially identified as an ASIC channel modulator, recent studies suggest its potential to inhibit TRPA1 activity. Hcr 1b-1 may reduce neuronal hyperexcitability induced by proinflammatory stimuli [82].

## ASIC channel modulators

Acid-sensing ion channels (ASICs) are sodium-selective (Na⁺) channels that belong to the epithelial sodium channel/degenerin (ENaC/DEG) superfamily. These voltage-independent channels are found in both the central and peripheral nervous systems of vertebrates and have been suggested to play a crucial role in pain perception under pathological conditions such as inflammation and ischemia [83]. ASICs are also essential for various organisms due to their pH sensitivity, which facilitates ion passage and enables diverse cellular functions. As a result, some venomous organisms have evolved modulators that affect these channels, incorporating them into their venom's toxic components. In cnidarians, several ASIC channel modulators have been identified. For instance, the 42-residue peptide APETx-2, isolated from the sea anemone *Anthopleura elegantissima*, was the first selective inhibitor of ASIC3 channels [58]. Similarly, the peptide Hcr 1b-1, isolated from *H. crispa*, is an APETx-2 analog with lower potency but potentially comparable effects on ASIC3 channels [82]. Other ASIC-modulating toxins are Ugr 9-1 and PhcrTx-1, derived from the sea anemones *Urticina grebelnyi* and *Phymanthus crucifer*, respectively. Ugr 9-1 acts by blocking ASIC3 channels, while PhcrTx-1 inhibits transient currents in rat sensory neurons [58,84].

## Small cysteine-rich peptides

**Figure 5.**
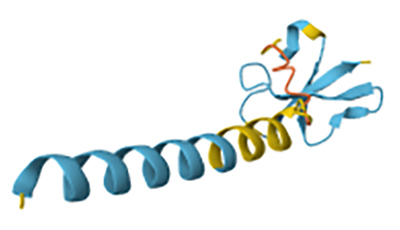
Predicted 3D-structure of a small cysteine-rich protein (SCRiP) from the coral *A. millepora* (UniProt ID: C1KIY9). The structure, predicted by AlphaFold v2.0, highlights conserved α-helical elements and cysteine-stabilized motifs characteristic of cnidarian SCRiPs. Image courtesy of Jumper et al. [72] and Varadi et al. [73].

Small cysteine-rich peptides (SCRiPs) represent the first major family of toxins detected in stony corals. These peptides consist of a hydrophobic N-terminal signal peptide and a C-terminal cysteine-rich domain (Figure 5). They were first discovered through an *in silico* search for antimicrobial peptides (AMPs) in the stony corals *Orbicella faveolata*, *Montipora capitata*, and *Acropora millepora*. However, they were not classified as AMPs but rather as peptides involved in the calcification process of these reef-building corals during thermal stress [85,86]. SCRiPs can be classified into four monophyletic clades based on sequence identity: SCRiP-α, comprising 84 sequences from stony corals of the *Acroporidae* family; SCRiP-β, consisting of 45 sequences from over 15 species across seven coral families, predominantly within *Merulinidae*; SCRiP-γ, containing 32 sequences from more than 18 species spanning four coral families, including *Acroporidae*; and SCERiP-δ, which includes 31 sequences, with 19 from sea anemones (superfamilies *Actinoidea* and *Metridioidea*) and 12 from stony corals of the genus *Acropora* [86].

In this context, one of the first studies providing evidence of the toxic activity of SCRiPs was conducted by Jouiaei et al. [87]. In that study, the recombinant peptides Amil_SCRiP2 and Amil_SCRiP3, identified in *A. millepora*, were obtained. These peptides were proposed as potential neurotoxins, as their injection into zebrafish (*Danio rerio*) induced a progressive series of toxic effects, including frequent spasms and shivering, loss of tactile response, and ultimately complete paralysis. All fish exposed to Amil_SCRiP2 died within 200 minutes post-exposure, while Amil_SCRiP3 led to mortality within 16 hours. These findings support the hypothesis that SCRiPs may function as neurotoxins [87]. Additionally, studies by Logashina et al. [88] and Schmidt et al. [51] have provided new evidence reinforcing the hypothesis that SCRiPs represent a novel family of cnidarian toxins. In these studies, the peptides Ueq 12-1, Hact-4, and Hact-SCRiP1 were isolated from *Urticina eques* and *Heliofungia actiniformis*, respectively. In both cases, the peptides exhibited a β-defensin-like fold, further supporting their connection to SCRiPs [51,88].

## Pore forming-toxins

Pore-forming toxins (PFTs) are common components of cnidarian venoms, acting by penetrating cell membranes and facilitating the diffusion of small molecules and solutes, ultimately leading to osmotic imbalance and cell lysis [89]. These toxins exhibit a dual structural state: a water-soluble monomeric form that binds to target cell receptors, and an oligomeric membrane-bound form that assembles into integral pores in the target cell [90]. Based on their secondary structure and membrane penetration mechanism, cnidarian PFTs are classified into two types: α-PFTs, which are rich in helices and form α-helical barrel pores, and β-PFTs, which are β-sheet-rich and assemble into β-barrel pores [12]. A wide variety of PFTs have been identified in cnidarian venoms, including actinoporins, jellyfish PFTs, and hydralysins.

**Figure 6.**
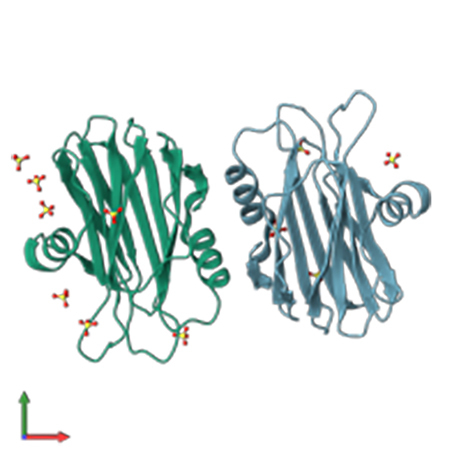
Crystal structure of Equinatoxin II from the sea anemone *A. equina*, resolved by X-ray diffraction at a resolution of 1.9 Å. Image adapted from PDBe (PDB ID: 1IAZ). Courtesy of Athanasiadis et al. [98].

Actinoporins, found in anthozoans, belong to the α-PFT family and have a molecular mass ranging from 18 to 20 kDa. These toxins specifically interact with sphingomyelin or phosphatidylcholine and are capable of inducing hemolysis, cardiovascular arrest, and cytotoxicity [91-93]. Examples include sticholysins I and II from *S. helianthus* [94] and equinatoxins I-V from *A. equina* (Figure 6) [95-97].

Jellyfish PFTs, particularly those from cubozoans, are among the most potent and fast-acting toxins in the phylum Cnidaria. These toxins have a molecular mass ranging from 40 to 46 kDa and possess a combined α-helix and β-sheet structure, making them highly effective pore-formers that induce rapid cell death by lysing membranes [99,100]. Notable examples include CFTX1 and CFTX2 from *C. fleckeri*, which cause cardiotoxicity and cytotoxicity [101]. Another group of PFTs identified in cnidarians includes hydralysins, which belong to the β-PFT family and were discovered in the endodermal digestive cells of *Hydra viridissima* [102]. These toxins are thought to play a role in prey digestion due to their cytolytic activity [102-104].

## Small molecule toxins 

Cnidarian venoms have been primarily studied for their proteinaceous toxins, such as those described above. However, bioactive non-protein compounds have also been identified, playing a significant role in the biological effects of these venoms. For example, 5-hydroxytryptamine (5-HT) from *H. viridissima* and histamine found in the venoms of *A. viridis* and *A. equina* induce acute pain and increase vascular permeability, serving both defensive functions and enhancing the activity of other venom components [103,105,106].

Additionally, bunodosine (an N-acylamino acid) and caissarone (a quaternary purine derivative) have been purified from *B. cangicum* and *B. caissarum*, respectively. These compounds exhibit potent analgesic activity and intestinal function stimulation [107,108]. Another example of a bioactive non-protein toxin is palytoxin, isolated from soft corals of the genera *Palythoa* and *Zoanthus*. This toxin interacts with the Na⁺/K⁺-ATPase pump, triggering massive membrane depolarization and tissue contraction [109]. Furthermore, a non-protein fraction of the venom from the hydrocoral *M. complanata* contained polyoxygenated alkylbenzenes capable of inducing seizures and rapid death in mice [110]. Several of the cnidarian toxins previously described have been identified, characterized, or functionally annotated through omics approches. Transcriptomic and proteomic analyses have proven especial value in revealing enzymes such as PLA2s and metallopeptidases in species like *P. hydriforme*, *Cyanea* sp., and *R. esculentum*, while genomic studies have provided insights into the diversity and evolutionary origin of neurotoxins and Kunitz-type inhibitors. These technologies have also played a key role in detecting novel toxin families, such as SCRiPs, and in elucidating the biosynthetic origin of non-protein molecules. In the following section, we explore how omics approaches have enabled such discoveries and discuss their relevance for bioprospecting and therapeutic development.

## Omics approaches in the identification of toxic compounds from cnidarians with potential therapeutic applications

The identification and characterization of cnidarian toxins have been significantly advanced by omics technologies. These approaches, including genomics, transcriptomics, and proteomics, have enabled the discovery of novel toxin-coding genes, elucidated their expression profiles, and facilitated the annotation of putative venom components across diverse cnidarian species. Omics approaches have enabled the identification of key venom components across cnidarians, including PLA2s and metallopeptidases in *P. hydriforme*, *Cyanea* sp., *B. plumatellae*, and *R. esculentum* [26,27,56], as well as Kunitz-type peptides and ion channel modulators like APETx-2 and Hcr 1b-1 in sea anemones [69,93]. These findings underscore the central role of omics tools in uncovering the molecular complexity of cnidarian venoms and their potential biomedical applications.

The study of cnidarian toxins has revealed a wide range of compounds with potential therapeutic applications, including peptides, proteins, and non-protein bioactive molecules that affect various biological systems. However, the complexity and diversity of cnidarian venoms present significant challenges for their identification and characterization. To address these challenges, advanced analytical approaches have been developed, enabling a more precise and detailed examination of venom composition. Omics sciences have emerged as essential tools for the comprehensive analysis of these venoms, facilitating the identification of novel components, the investigation of their mechanisms of action, and the exploration of their pharmaceutical potential [5,111,112].

Omics sciences encompass a set of advanced methodologies that utilize high-throughput technologies to analyze biological systems comprehensively. Disciplines such as genomics, transcriptomics, proteomics, and metabolomics enable the identification, quantification, and characterization of key biomolecules, including genes, messenger RNA, peptides, proteins, and metabolites. These approaches provide a detailed molecular analysis of organisms, allowing for unprecedented comprehensive studies of the molecular components of cnidarian venoms [113]. Their broad scope and applicability have transformed bioactive compound research, opening new opportunities for the discovery and characterization of molecules with therapeutic potential [114].

Marine ecosystems, in particular, represent an invaluable source of bioactive compounds due to their vast biodiversity. The application of omics techniques not only facilitates the exploration of this biological wealth but also enables its utilization in the development of novel treatments and biotechnological products. In this context, cnidarian venoms constitute a promising and largely unexplored resource, as they contain a wide diversity of toxins that have evolved to specifically interact with the molecular systems of their prey [12]. The identification and characterization of these toxins through omics sciences not only enhance our understanding of the biological effects of venoms but also open new avenues for the development of innovative drugs for conditions such as chronic pain, neuromuscular disorders, and inflammatory diseases. This underscores the significance of cnidarians as a valuable source of bioactive molecules with biomedical potential [5,115].

## Genomic studies

GenBank contains approximately 46 cnidarian genomes, enabling the identification of gene families encoding key toxins [5]. For instance, in cnidarians of the genus *Palythoa*, such as *Palythoa mizigama* and *Palythoa umbrosa*, genes associated with putative toxins have been identified and classified into six groups based on their mechanism of action: neurotoxins, hemostatic and hemorrhagic toxins, peptidase inhibitors, membrane-active peptides, enzymes with mixed functions, and allergenic peptides (innate immunity modulators). Notably, the gene encoding palytoxin (PTX) has been detected in multiple *Palythoa* species [116].

Three TTL gene families encoding sea anemone toxin 8, hydrolase AB, and structural class 9a sea anemone toxin were found in *Exaiptasia pallida*, while two TTL gene families were identified, encoding CRISP and DNase II in *Hydra vulgaris* and another two TTL gene families coding for latrotoxin-like toxins and venom metallopeptidase (M12B) were detected in *O. faveolata*. Additionally, five TTL gene families were identified, encoding phospholipase A2, multicopper oxidase, peptidase M12A, snaclecs (C-type snake venom lectins), and actinoporins in *E. pallida*, *H. vulgaris*, and *O. faveolata* [60]. Another genomic study showed the presence of a gene family encoding peptides similar to conopeptide P in *Stylophora pistillata*. This class of peptides has therapeutic potential, as they act as competitive antagonists of neuronal and muscular nicotinic acetylcholine receptors (nAChRs), demonstrating analgesic effects in animal models [60,117].

Similarly, 127 toxin-associated genes have been identified in *R. esculentum*, including 60 metallopeptidase-encoding genes, 18 phospholipases, 13 nucleases and nucleotidases, 13 peptidases and inhibitors, 12 toxin-related genes, and 11 other venom-associated genes. Metallopeptidases are known to interfere with blood coagulation and induce necrosis, while phospholipases can cause hemolysis. Additionally, a detailed genomic analysis of this jellyfish revealed two novel toxin-associated genes: reticulocalbin, which plays a role in prey incapacitation by binding to Ca²⁺, and lysosomal acid phosphatase, which is involved in allergic reactions [28].

A genomic study on jellyfish revealed a diverse array of toxin-encoding genes, including aurelina (*A. aurita*), CbTX-I and CbTX-II (*Carukia barnesi*), CaTX-A and CaTX-B (*Carybdea alata*), CfTX-1, CfTX-2, CfTX-A, and CfTX-B (*C. fleckeri*), CqTX-A (*Chiropsalmus quadrigatus*), and MkTX-A and MkTX-B (*Malo kingi*) [118]. The therapeutic potential of cobratoxin (CbTX) as an analgesic has been previously proposed, as it has demonstrated antinociceptive effects in neuropathic pain rat models by activating nicotinic acetylcholine receptor (nAChR) α7 [119]. Additionally, in an *in silico* study using the Protein Families database seven precursor genes encoding ShK-like peptides were identified in the genome of *N. nomurai* [25,120]. *In vitro* assays in HEK cells showed that the peptide NnK-1 of *N. nomurai* can block voltage-gated potassium channels hKv1.3, hKv1.4, and hKv1.5. Given that the activation of Kv1.3 channels in human T and B lymphocytes is associated with autoimmune diseases, NnK-1 holds therapeutic potential for treating such conditions [121].

DNA sequencing allowed the identification of toxin-encoding genes in *T. stephensoni*, including those for ShK-like toxins, peptidase M12A, phospholipase A2 (PLA2), and other putative toxins. Similarly, *Actinia tenebrosa* was found to possess genes encoding putative toxins resembling insulin-like growth factor-binding protein (IGFBP) and Factor V-like toxins [122]. Notably, the ShK toxin, a potent blocker of voltage-gated potassium channel Kv1.3, has demonstrated therapeutic efficacy in animal models of human autoimmune diseases such as multiple sclerosis and rheumatoid arthritis. Moreover, ShK-186, also known as dalazatide, has successfully completed phases Ia and Ib of clinical trials [123].

Another genomic study carried out on the sea anemone *N. vectensis* demonstrated that the locus of the gene encoding type 2 ShK-like proteins is conserved. Additionally, in that study a model for genes encoding type 1 and type 3 ShK-like proteins was proposed, which was particularly novel since no model had previously been established for these genes. This finding highlights that genomics not only facilitates the identification of cnidarian toxin-encoding genes with pharmacological potential but also enables the construction of gene models for sequences that remain incomplete [124].

The study by Gacesa et al. [125], based on homology searches using BLAST, showed that out of the 55 toxins potentially produced by *Acropora digitifera*, 36 originated through duplication of their encoding genes. This finding indicates that gene duplication has played a key role in the diversification of toxins in this species. Gene duplication allows new gene copies to evolve, acquiring novel functions or specializing in specific roles, such as the production of toxins with distinct biological purposes (e.g., defense against predators). Thus, genomics plays a crucial role in identifying the evolutionary mechanisms underlying the diversification of toxin-encoding genes in cnidarians, which, in turn, may provide insights into the biological functions of these toxins and their therapeutic potential [125].

Another study, based on tBLAST searches, led to the identification of clusters of six actinoporin-like toxin-encoding genes in five sea anemone species: *N. vectensis*, *Stomphia coccinea*, *Epiactis japonica*, *H. crispa*, and *Diadumene leucolena* [126]. In a related study, Liew et al. [127] reported the presence of a family of six actinoporin-encoding genes in the genome of *Hydra magnipapillata* and highlighted a potential application of these proteins in immunotoxin therapy. In this approach, an actinoporin can function as an anticancer agent by forming a pore in the target cell membrane when fused to an antibody specific to that cell type.

Finally, Macrander et al. [128] found that among the toxin-encoding genes present in cnidarians, those associated with NaTxs and KTxs of types I and III appear to be specific to the order *Actiniaria*. Next-generation sequencing (NGS) genomic techniques have been instrumental in identifying the majority of toxin-encoding genes in sea anemones. Additionally, the authors used tBLAST to identify candidate toxin-encoding genes in actiniarians, including ASIC toxins (*H. crispa*), acrorhagins (*A. sulcata*, *H. crispa*, and *Megalactis griffithsi*), AETX-like toxins (*A. sulcata*), class 9a KTx (*A. sulcata*), and KTx types I (*A. sulcata*, *H. crispa*, and *M. griffithsi*), II (*A. sulcata*, *H. crispa*, and *M. griffithsi*), III (*A. sulcata*, *H. crispa*, and *M. griffithsi*), and V (*A. sulcata* and *H. crispa*). The study also identified metallopeptidases (*A. sulcata*, *H. crispa*, and *M. griffithsi*), membrane attack complex/perforin (MACPF) proteins (*A. sulcata* and *H. crispa*), EGF-like toxins (*A. sulcata* and *H. crispa*), and PLA2 (*A. sulcata*, *H. crispa*, and *M. griffithsi*) [128].

## Transcriptomic studies

Transcriptomic studies have significantly expanded our understanding of the diversity and function of toxin genes in cnidarians. Currently, more than 120 cnidarian transcriptomes are available in the GenBank and Transcriptome Shotgun Assembly databases, facilitating the identification of a wide range of toxin-related genes [129,130]. For instance, the analysis of 14 transcriptomes from cnidarians of the order *Actiniaria* (sea anemones) identified 39 toxin-encoding gene families, including Kunitz-type peptides, PLA2, sea anemone toxin 8, NaTx, and KTx [60]. Kunitz-type peptides exhibit broad therapeutic potential, functioning as analgesics and antiepileptics, as well as displaying anticoagulant activity by inhibiting the procoagulant peptidases factors VIIa and Xa [74]. Additionally, these peptides exert anti-inflammatory effects by blocking voltage-gated potassium channels of the Kv1.3 type, which are present in central nervous system cells [131]. This biological activity has been proposed for therapeutic applications in treating neurodegenerative disorders associated with chronic inflammation, such as Alzheimer’s and Parkinson’s disease [75].

A transcriptomic study of *M. alcicornis* led to the identification of transcripts encoding putative toxins, including neurotoxins (latroinsectotoxin, α-latrocrustotoxin, α-latrotoxin, turripeptides), metallopeptidases (astacin-like metallopeptidase, ADAMs), homeostasis-disrupting toxins (prothrombin activator, rincolin), serine peptidases, complement-affecting toxins, cysteine-rich venom proteins, phospholipases, phosphodiesterases, pore-forming toxins, and L-amino acid oxidases, among others [40]. Particularly, turripeptides are capable of inhibiting nAChRs α7 and α3β2, making them potential therapeutic agents for neurological disorders involving these receptors, such as Alzheimer’s and Parkinson’s disease, schizophrenia, a genetically transmissible form of epilepsy, inflammation, chronic pain syndromes, and myasthenia gravis [132]. Additionally, serine peptidases can prevent platelet aggregation and induce fibrinolysis. Certain kallikrein-type serine peptidases, such as KLK3, are involved in prostate cancer progression and metastasis. These therapeutic effects have recently drawn researchers' attention to serine peptidases as potential targets for treating this pathology [133].

A transcriptomic study of *M. complanata* identified 190 putative toxins, which were categorized based on their function into enzymes (including metallopeptidases, phospholipases, and lipases), hemostasis-disrupting toxins, pore-forming toxins, complement-disrupting toxins, among others. Notably, that study highlighted a *M. complanata* hemolysin-like protein (Mc_hemolysin-like) and a *M. complanata* hydralysin-like toxin (Mc_hydralysin-like), which exhibited sequence similarity to four centipede toxins (TX14A_SCODE, A0A646QER6_9MYRI, A0A646QI04_9MYRI, and A0A646QD69_9MYRI) and four hydralysins (HLYS_HYDVU, HLYS1_HYDVU, HLYS2_HYDVU, and HLYS3_HYDVU), respectively. Additionally, a virtual screening for antimicrobial peptides (AMPs) was conducted, yielding 1,966 and 3,876 matches in AMP databases such as APD3 and DRAMP, respectively. Most matches exhibited sequence similarity to SK84, cgUbiquitin, Ubiquicidin, TroTbeta4, SPINK9-v1, and histone-related antimicrobial peptides [41].

Similarly, a transcriptomic study of cnidarians from the family Myxobolidae, specifically *Thelohanellus kitauei*, *Myxobolus xiantaoensis*, *Myxobolus ampullicapsulatus*, *Myxobolus turpisrotundus*, and *Myxobolus honghuensis* showed the expression of toxin-like protein (TLP) encoding genes. These proteins were classified based on their biological function into neurotoxins (Kunitz, CRISP), cytolysins (actinoporins), peptidase inhibitors (cystatin), hemorrhagic toxins (metallopeptidases, peptidase S1, and true venom lectins), allergens (AB-hydrolase), and enzymes (phospholipase A2, glycosyl hydrolase 56, and PDGF/VEGF) [134]. Notably, actinoporins are characterized by their ability to bind to cell membranes and form pores. These toxins have attracted therapeutic and biotechnological interest for applications in immunotoxin design, nanopores, and adjuvants, among others [135].

A transcriptomic study of the cnidarian *Zoanthus natalensis* (family *Zoanthidae*) demonstrated the expression of genes encoding neurotoxins, hemostatic and hemorrhagic toxins, peptidase inhibitors, allergenic toxins, and venom auxiliary proteins [58]. The toxin groups identified in this species closely resemble those reported by Xiao et al. [134] in cnidarians of the family Myxobolidae [134]. Marine venoms have been recognized as an emerging source of peptide-based drugs with therapeutic potential, including analgesics, anticancer agents, and treatments for neurological disorders [136]. For example, the peptide APETx2, produced by *A. elegantissima*, has demonstrated analgesic effects in inflamed or ischemic tissues under acidic conditions by inhibiting ASIC3 channels. Its homolog, APETx4, has been shown to activate voltage-gated potassium channels [58].

Similarly, through a transcriptomic study the expression of genes encoding neurotoxins (ShK domain, cysteine-rich venom protein, Kazal_1 domain-containing turripeptide), hemostatic and hemorrhagic toxins (prothrombin activator F5/F8 type C, trypsin-like prothrombin activator, rincolin, coagulation factors V and X), and enzymes with mixed functions (acetylcholinesterase, L-amino acid oxidase, phospholipase A2, putative endothelial lipase, putative lysosomal acid lipase, and venom phosphodiesterase) were identified in *Pachycerianthus cf. maua*, *Pachycerianthus borealis*, *Isarachnanthus nocturnus*, and *Ceriantheomorphe brasiliensis*. That same study showed the presence of peptidase inhibitors (Kunitz-type serine peptidase inhibitors, tenitoxin U24-Pn1a), allergenic and innate immunity-modulating toxins (venom allergen, venom serine peptidase, and venom peptidase), and auxiliary proteins (astacin-like metallopeptidase toxin, nematocyst-expressed protein, reticulocalbin, and neprilysin) [39].

In this context, genomic and transcriptomic studies have been of considerable value for identifying genes or transcripts that encode toxins with significant therapeutic potential. The identification of genes involved in toxin biosynthesis paves the way for their subsequent adaptation to heterologous expression systems, a critical step for large-scale production in more accessible hosts. This strategy not only enhances production efficiency but also facilitates purification and enables their application in the development of innovative therapies, such as toxin-conjugated antibodies or therapeutic recombinant proteins, as discussed later in this article.

## Proteomic studies

Proteomic studies have significantly expanded our understanding of protein diversity and function in cnidarians, enabling in-depth investigation of the toxic components of their venoms. Approximately eight nematocyst-specific proteomes have been recorded in databases such as ProteomeXchange and PRIDE, while other databases provide additional insights into the expression of proteins involved in toxicity [137]. For instance, a proteomic analysis of the venom from the jellyfish *R. esculentum* and *Sanderia malayensis* showed the presence of 40 and 51 putative toxins, respectively, with peptidases being the most prevalent in both species (60%) [56]. Similarly, Tassara et al. [43] analyzed the proteome of the blue jellyfish *V. velella*, leading to the identification of 783 proteins associated with structural components, enzymes, and putative toxins. Among the latter, 15 different classes of toxic compounds were identified, including peptidases (disintegrin and metalloproteinase), phospholipases (PI-phospholipase C-like phospholipase D1), anticoagulant proteins (urokinase-type plasminogen activator), peptidase inhibitors (Kunitz-type peptidase inhibitor-1 and papalysin-1), neurotoxins (Ly-6/1-like neurotoxin, OH-55 long neurotoxin, and kappa-stichotoxin-She3a), and pore-forming toxins (PFT XaxB), among others [43].

Similarly, a proteomic study of the tentacles and mucus of *Anthopleura dowii* showed 156 polypeptides, of which 48 were exclusive to mucus, 20 were found only in tentacles, and 88 were present in both samples. Among these, 23 polypeptides were associated with venom, including 17 belonging to toxin families such as Na⁺ channel inhibitor toxins (Delta-actitoxin-Avd1e1, Ael1b, Axm1f, Ael1c, and Axm1a), Kunitz/Kv2-type peptides (*KappaPI-actitoxin-Ael3a*), and peptidase inhibitors or peptidases [138].

Mazzi Esquinca et al. [139] characterized the proteomic composition of the tentacles and mucus of *B. caissarum*, a sea anemone found along the Brazilian coast. Their proteomics analysis allowed detection of 430 polypeptides, of which 316 were abundant in tentacles and 114 in mucus. Among these, 23 showed similarity to previously characterized toxins, including sea anemone types 1, 2, and 3 toxins that target potassium channels, EGF domain peptides, Kazal-type serine peptidase inhibitors, sea anemone toxin 8, astacin domain proteins, and cystatins, among others. Similarly, Li et al. [140] conducted a proteomic study on the venom composition of the sea anemone *H. magnifica*. In that study 101 toxins were identified, including 79 proteins and 22 peptides, which were classified using the UniProt database. The identified components included 36 functional proteins, 26 peptidases, 23 neurotoxins, 9 peptidase inhibitors, 5 innate immunity and allergenic proteins, 1 pore-forming toxin, and 1 hemostatic or hemorrhagic protein.

Hernández-Elizárraga et al. [141] conducted a comparative proteomic study on the soluble proteome of normal and bleached specimens of the hydrocoral *M. complanata* exposed to the 2015-2016 El Niño-Southern Oscillation in the Mexican Caribbean. That analysis revealed 35 proteins with differential abundance in bleached specimens, classified into eight categories, including primary metabolism, DNA repair, cytoskeletal components, signaling proteins, and toxins. Among the toxin-related proteins, four were identified with similarity to known toxins: secreted acidic PLA2 PA4, ecotoxin-2, DELTA-actitoxin-Oor1b, and an astacin-like metallopeptidase toxin 5. Similarly, Olguín-López et al. [142] analyzed the differential proteomic profile of *M. alcicornis* from the Mexican Caribbean in response to bleaching. The study identified 17 proteins with differential abundance, including key regulators of calcium homeostasis, cytoskeletal organization, and putative toxins such as a metallopeptidase, phospholipase A2, and DELTA-actitoxin-Ate1a. Additionally, a mass spectrometry-based analysis of the soluble proteome from *M. alcicornis* nematocysts revealed proteins with sequence homology to metallopeptidases (zinc metalloproteinase), pore-forming toxins (DELTA-actitoxin-Aeq1b), and neurotoxins (CrTx-A), highlighting the complexity of toxin biosynthesis in this hydrocoral [40].

## Metabolomic studies

Metabolomic studies in cnidarians are limited and have primarily focused on developing conservation and restoration strategies for these marine organisms. However, they have also been used to analyze the variability and function of venom metabolites to identify novel compounds with therapeutic potential. For instance, a metabolic profiling study of sea anemones and jellyfish exposed to high temperatures and UV radiation identified a cytotoxic macrolide, Salarin B, in *Entacmaea quadricolor* and *Cassiopea andromeda*. Additionally, a cytotoxic diterpenoid called Brasicolen was detected specifically in *E. quadricolor*, demonstrating efficacy against lung adenocarcinoma and lymphoma [143-145]. Moreover, polyacetylenocarboxylic acids, also known as montiporic acids, have been identified in corals of the genus *Montipora*. These compounds exhibit both cytotoxic and antimicrobial activity [146,147].

Santacruz et al. [148] conducted a study on soft corals from the Colombian Caribbean, identifying the diterpene 13-keto-1,11-dolabell-3(E),7(E),12(18)-triene in the coral *Pseudoplexaura flagellosa*. This metabolite exhibited cytotoxic activity against SiHa (human cervical carcinoma) and A549 (human alveolar lung carcinoma) cell lines, with IC50 values of 0.03 µg/mL and 0.02 µg/mL, respectively [148]. Additionally, the same research group performed a comparative metabolomic study on *Plexaurella* spp., identifying asperdiol and plexaurolone, both of which demonstrated cytotoxic activity against the PC3 (human prostate carcinoma) cell line [149]. 

Metabolomic analyses of corals from the genera *Sarcophyton*, *Sinularia*, *Eunicea*, and *Clavularia* have identified various cembranoid diterpenes, such as sarcophine, sarcophytolide, and sarcophytolide B or C. These metabolites exhibit anti-inflammatory, cytotoxic, and antibacterial activities [150]. Similarly, Hegazi et al. [151] analyzed the secondary metabolome of soft corals from the Egyptian Red Sea, identifying oxysterols in *Sinularia leptoclados*, *Sarcophyton roseum*, and *Sarcophyton acutum*, which have demonstrated cytotoxic activity.

These findings underscore the importance of omics sciences in identifying toxins and other bioactive compounds found in the venoms of cnidarians. However, despite the significant interest in these compounds due to their potential applications, their study and purification face considerable challenges. First, many of these species are protected, making it difficult to obtain permits for their collection and investigation. Additionally, sampling, storage, and transportation require highly specific conditions, as any variation can compromise the integrity of the biological material. Moreover, the limited amount of tissue in these organisms restricts the extraction of sufficient quantities for the thorough purification of individual components. These limitations highlight the need to develop more efficient and sustainable methods to harness the bioactive potential of these species without compromising their conservation [6]. Table 2 summarizes the main bioactive non-protein compounds identified in cnidarian venoms.

**Table 2.** Small molecule toxins in cnidarian venom.

Polyketides		
Bunodosine	*Bunodosoma* spp.	[107]
Caissarone	*Bunodosoma* spp.	[70]
Palytoxin	*Palythoa* spp & *Zoanthus* spp.	[109]
Polyoxigenated alkylbenzenes	*M. complanate*	[110]
Salarin B	*E. quadricolor* & *C. andromeda*	[145]
Montiporic acids	*Montipora* spp.	[146]
Asperdiol	*Plexaurella* spp.	[149]
**Terpenoids**		
Brassicolene	*E. quadricolor* & *C. andromeda*	[145]
13-keto-1,11-dolabell-3(E),7(E),12(18)-triene	*P. flagellosa*	[148]
Plexaurolone	*Plexaurella* spp.	[149]
Oxysterols	*S. leptoclados, S. roseum* & *S. acutum*	[151]
Sarcophine	*Sarcophyton* spp.*, Sinularia* spp., *Eunicea* spp., & *Clavularia* spp.	[150,151]
Sarcophytolide	*Sarcophyton* spp.*, Sinularia* spp., *Eunicea* spp., & *Clavularia* spp.	[150,151]
Sarcophytolide B and C	*Sarcophyton* spp.*, Sinularia* spp., *Eunicea* spp., & *Clavularia* spp.	[150,151]
5,8-epidioxysteroids	*Condylactus* spp.	[152]
Cladielloide B	*Cladiella* spp.	[153]
3,4-epoxy-nephthenol acetate	*Nephthea* spp.	[154]
Sangiangol A and B	*Anthella* spp.	[155]

## Integration of omics sciences in the discovery of toxic compounds in cnidarian venoms

The study of cnidarian venoms has undergone a significant transformation with the incorporation of omics technologies. Currently, there is a growing need to adopt integrative approaches that enable a comprehensive analysis of the composition, function, and evolution of toxic compounds in these biological systems. In this context, the combination of transcriptomic, genomic, proteomic, and metabolomic data - an approach known as multi-omics - has proven to be essential for the discovery of novel toxins, particularly in understudied organisms such as cnidarians.

High-throughput transcriptomics (RNA-seq) has enabled the detection of toxin genes expressed in secretory tissues, even in small organisms or those that are difficult to maintain alive under laboratory conditions [156]. This approach is often integrated with proteomic analyses to confirm the presence of the corresponding protein products. Transcriptomic data are further enriched through genomics, using genome-guided assemblies and functionally annotated databases enhanced by proteomic evidence. This synergy has facilitated the identification of toxin gene families, the analysis of gene duplication events, and the evolutionary reconstruction of toxic genes from non-toxic precursors [157,158]. The inclusion of metabolomic data adds an additional layer of insight, allowing the assessment of not only the presence but also the functional role of small bioactive molecules involved in venom modulation and its physiological effects [158,159].

Beyond these consolidated platforms, emerging technologies now provide a spatial dimension to venom molecular analysis. One of the most notable is mass spectrometry imaging (MSI), an untargeted technique that enables the mapping of toxin distribution in tissues without prior knowledge of their identity. Using modalities such as MALDI-MSI, researchers can generate two-dimensional maps of toxin abundance across histological sections, revealing differential distribution patterns in various organisms, including reptiles, arthropods, and cnidarians [158,160,161]. Furthermore, functional applications such as functional MSI (fMSI) allow the *in situ* detection of enzymatic activities, including those associated with PLA2s [162].

An illustrative case is *Anthopleura cascaia*, in which three serine peptidase inhibitor peptides (ACPI-I, ACPI-II, and ACPI-III) were identified through mass spectrometry coupled with functional assays. Their spatial distribution, determined by MSI, revealed specific localization in tentacles, pedal disc, and mesenteries, suggesting distinct roles in defense, digestion, and prey capture. These findings not only expand the toxin repertoire of the genus *Anthopleura* but also validate MSI as an effective tool for linking the structure, function, and tissue localization of toxins in organisms lacking centralized venom glands [163].

In parallel, spatial transcriptomics (ST) represents a major technological advancement by enabling the precise localization of toxin transcripts within their native tissue context. This technique involves tissue sections mounted on slides containing spatially barcoded poly-T probes that capture mRNAs to generate cDNA libraries with positional information [156]. Unlike other methods, ST does not require pooling of small samples - thereby preserving statistical power - and minimizes contamination from non-venom-producing tissues. Its high sensitivity allows spatial resolutions of up to 55 µm (equivalent to 5-10 cells), making it especially suitable for organisms with reduced or poorly defined anatomical structures [156,164]. When combined with single-cell transcriptomics, ST enables the accurate identification and localization of cell types involved in toxin synthesis and secretion.

**Figure 7.**
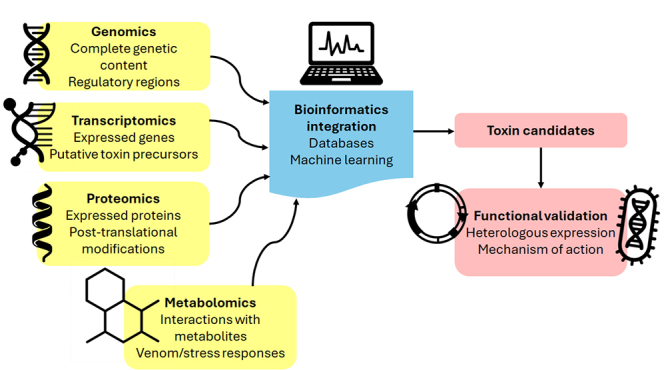
Schematic representation of omics integration for the discovery and characterization of cnidarian toxins. Genomic, transcriptomic, proteomic, and metabolomic data contribute complementary layers of information to bioinformatics pipelines that combine database mining and machine learning. This approach enables the identification of toxin candidates, which are subsequently validated through heterologous expression and functional assays to determine their mechanism of action.

Altogether, the integration of multi-omics approaches with spatial technologies has revolutionized the field of venomics. These advancements not only facilitate the identification of novel toxins with therapeutic potential but also provide deeper insight into the regulatory, biosynthetic, and functional mechanisms operating within venom-producing systems. Such methodological innovations pave the way for a more efficient and targeted discovery of toxic compounds with biomedical and biotechnological applications. In the following section, we explore how the data generated through omics studies has been leveraged to produce cnidarian toxins recombinantly, enabling detailed evaluation of their bioactivity and biomedical potential (Figure 7).

## Heterologous expression of cnidarian toxins

**Figure 8.**
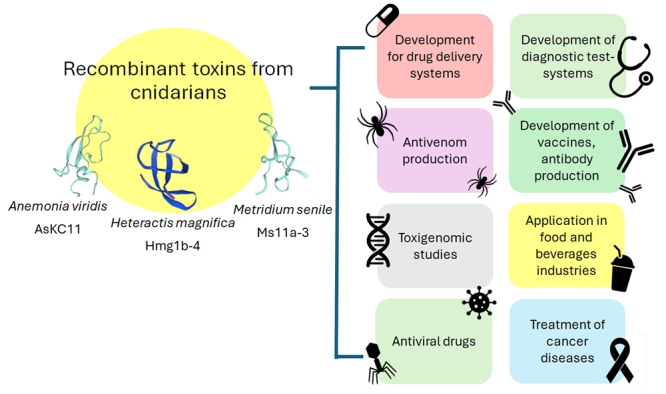
Biomedical and biotechnological applications of recombinant cnidarian toxins. Structural models of recombinant toxins from *A. viridis* (AsKC11), *H. magnifica* (Hmg 1b-4), and *M. senile* (Ms11a-3) are shown, alongside their potential applications in drug delivery systems, diagnostics, vaccine development, antivenom production, food industry, and cancer/antiviral treatments. Image adapted and modified from Efremenko et al. [166] published under a Creative Commons Attribution License (CC BY 4.0).

The study of toxins derived from venomous animals, particularly cnidarians, has revealed that their venoms are complex mixtures of peptides and proteins with remarkable biological activity. These molecules are essential for understanding envenomation mechanisms and exploring their pharmaceutical and biotechnological applications. Heterologous expression of toxins has emerged as a key tool in this field, enabling not only the production of sufficient quantities for in-depth studies but also genetic manipulation to engineer variants with specific properties. With the increasing availability of genomics and transcriptomics data, it is now possible to explore novel toxins with unique bioactive characteristics that may not naturally exist, expanding possibilities for therapeutic development and biotechnological applications. Furthermore, this approach reinforces the importance of developing sustainable methods to harness the bioactive potential of these toxins without compromising biodiversity (Figure 8) [6,165]. 

For instance, a proteomic study on *Cnidopus japonicus* identified peptides with novel cysteine-based structures, leading to the recombinant production of CjTL7, CjTL8, and AnmTx Cj 1c-1 in an *Escherichia coli* system. Toxicity assays revealed that the latter two recombinant toxins exhibited strong toxic effects on crustaceans and insects, respectively [167]. Another example is the study by An et al. [46] which involved the identification, characterization, and recombinant production of AsKC11, a novel modulator of G protein-coupled inwardly rectifying potassium (GIRK) channels found in the venom of the sea anemone *A. sulcata*. Recombinant AsKC11 was confirmed as a new GIRK channel activator.

Gladkikh et al. [49] demonstrated the anxiolytic, analgesic, and anti-inflammatory effects of the peptides Hmg 1b-2 and Hmg 1b-4, derived from *H. magnifica*. In this study, both peptides were heterologously expressed in *E. coli*. Recombinant Hmg 1b-4 exhibited a more pronounced anxiolytic and anti-inflammatory effect, while both peptides showed comparable analgesic activity. Similarly, the crude venom of *M. senile* was found to interact with nAChRs. This activity was attributed to four newly identified peptides (Ms11a-1 to Ms11a-4), which were successfully obtained through recombinant expression. Among them, Ms11a-3 exhibited the strongest antagonistic effect on nAChR α9α10 [54]. Kvetkina et al. [168] successfully obtained a recombinant pore-forming toxin identified in *H. crispa*. Using *E. coli* as an expression system, the recombinant toxin Hct-S3 was produced and exhibited cytotoxic activity against malignant cancer cells, including MDA-MB-231 breast cancer cells (IC50 = 7.3 μM), HT-29 colorectal cancer cells (IC50 = 6.8 μM), and SK-MEL-28 melanoma cells (IC50 = 8.3 μM). These results suggest that Hct-S3 could be a promising anticancer drug. Table 3 presents the main recombinant toxins identified in cnidarians.

**Table 3.** Recombinant toxins identified in cnidarians.

Toxin	Organisms of origin	Expression system	Reference
**Phospholipase**			
UcPLA2	*U. grebelnyi*	*E. coli*	[169]
**Metallopeptidases**			
NnM469	*N. nomurai*	*Pichia pastoris*	[120]
**Neurotoxins**			
SHTX-Sg1a	*Stichodactyla gigantea*	*E. coli*	[170]
SHTX-Sha1a	*S. haddoni*	*E. coli*	[170]
SHTX-Sm1a	*Stichodactyla mertensii*	*E. coli*	[170]
TLTX-Ca1a	*Cryptodendrum adhaesivum*	*E. coli*	[170]
TLTX-Hh1a	*Heterodactyla hemprichii*	*E. coli*	[170]
TLTX-Ta1a	*Thalassianthus aster*	*E. coli*	[170]
Crassicorin-I	*Urticina crassicornis*	*E. coli*	[171]
**Kunitz-type proteinase inhibitors**			
HGCS 1.20	*H. crispa*	*E. coli*	[172]
HGCS 1.19	*H. crispa*	*E. coli*	[173]
HGCS 1.36	*H. crispa*	*E. coli*	[173]
ShPI-1ª	*S. helianthus*	*P. pastoris*	[174]
AsKC11	*A. sulcata*	*E. coli*	[46]
**TRPV1 channel inhibitors**			
Tst2	*T. stephensoni*	*E. coli*	[48]
HCRG21	*H. crispa*	*E. coli*	[175]
**TRPA1 channel modulators**			
Ueq 12-1	*U. eques*	*E. coli*	[88]
**ASICs channel modulators**			
APETx-2	*A. elegantissima*	*E. coli*	[176]
Ugr 9a-1	*U. grebelnyi*	*E. coli*	[169]
Hmg 1b-2 & Hmg 1b-4	*H. magnifica*	*E. coli*	[49]
**Small Cysteine-Rich Proteins**			
Amil-SCRiP2 & Amil_SCRiP3	*A. millepora*	*E. coli*	[88]
**Pore-forming toxins**			
Esticolisina II	*S. helianthus*	*E. coli*	[177]
FraC	*Actinia fragacea*	*E. coli*	[178]
Hydralysin	*H. viridissima*	*E. coli*	[102]
Hct-A3, Hct-S3 & Hct-A5	*H. crispa*	*E. coli*	[179]
HALT-1	*H. magnipapillata*	*E. coli*	[180]
CJTOX-I & CJTOX-II	*Cribrinopsis japonica*	*E. coli*	[181]
Hct-S3	*H. crispa*	*E. coli*	[168]
Nigrelysin	*Anthopleura nigrescens*	*E. coli*	[182]
**Kazal-type serine peptidase inhibitor**			
CcKPI1	*C. capillata*	*E. coli*	[183]
**Inhibit nicotinic acetylcholine receptors**			
Ms11a-1, Ms11a-2, Ms11a-3 & Ms11a-4	*M. senile*	*E. coli*	[54]
**Others**			
CjTL7 & CjTL8	*C. japonicus*	*E. coli*	[167]
Anm Tx Cj 1c-1	*C. japonicus*	*E. coli*	[167]

The toxins classified under “Others” have not yet been fully characterized and therefore cannot be assigned to any of the eight categories established in this or other publications.

## Advances, challenges, and emerging strategies in the expression and production of cnidarian toxins 

The recombinant expression of cnidarian toxins is a fundamental tool for their functional and structural characterization as well as for their application in biotechnology and therapeutic development. However, the structural complexity and biochemical nature of many of these molecules, including cysteine-rich peptides requiring specific post-translational modifications (PTM), make their production in heterologous systems particularly challenging [184]. 

Bacterial systems, primarily *E. coli*, remain widely used due to their operational simplicity, low cost, and high productivity. Strategies such as periplasmic expression, the use of inclusion bodies, and co-expression (e.g. the CyDisCo system) have been developed to address the challenges associated with the correct folding of peptides containing multiple disulfide bonds [185,186]. Genetically engineered strains such as Shuffle® and Origami™ have also been employed. Nevertheless, the absence of enzymatic machinery for complex PTMs, such as glycosylation or amidation, limits the production of biologically active products [187,188]. Additionally, the requirement for refolding protocols to recover functional proteins can significantly impact final yields. 

 As an alternative, yeast systems, particularly *P. pastoris*, offer a more favorable eukaryotic environment for proper folding and the execution of certain PTMs [184]. This system has been successfully employed to express toxins such as APETX2 from sea anemones, as well as various peptidases and neurotoxins from other venomous animals [176]. Advantages of this system include high-density cultivation, stable gene integration, and efficient protein secretion into the medium [189]. However, challenges such as hyperglycosylation, proteolytic degradation, and incompatibility of glycosylation patterns with therapeutic applications have been reported. Engineered strains such as GlycoSwitch® and strategies like PEGlycation have been developed to overcome some of these limitations [189,190]. 

BEVS has been successfully used to produce difficult toxins. These systems also support protein secretion and the generation of stable cell lines [191]. However, their implementation is more complex than microbial systems, and expression levels depend on variables such as promoter selection, cell line, and signal peptide [184]. Recent innovations, including the BaculoDirect™ system and CRISPR/Cas9 genome editing, have significantly improved their efficiency and versatility [192]. 

At the other end of spectrum, mammalian cell expression systems offer the most physiologically relevant environment to produce toxins requiring highly specific PTMs, such as O- and N-glycosylation, hydroxylation, or γ-carboxylation [184]. Despite their high fidelity in folding and modification, these systems face major drawbacks including high cost, slow growth, and lower protein yields [184]. Nevertheless, systems such as HEK293 and CHO have proven useful for the production of cysteine-dense peptides (CDPs) and have been employed in high-throughput surface display, secretion, and mutagenesis studies [193,194].

Despite these advances, achieving efficient production of toxins with native-like structural and functional properties remains a challenge. The choice of expression system must consider the biochemical characteristics of the toxin, its intended applications, and the available purification strategies. In this context, additionally approaches are being explored to enhance recombinant toxin production.

## Tags and fusion proteins

A key component in optimizing the expression and purification of recombinant toxins is the use of peptide tags and fusion proteins, which improve solubility, promote disulfide bond formation, and simplify purification processes. 

Among the most used is the poly-His tag, consisting of six to ten histidine residues, which enables purification through immobilized metal affinity chromatography (IMAC) [195]. This tag is compatible with various host systems and tolerates denaturing conditions. However, its efficiency may be reduced in insect or mammalian cells due to the presence of endogenous histidine-rich proteins, requiring additional purification steps [184]. Alternatively, tags such as FLAG or c-Myc allow purification via specific antibodies under mild conditions, although they come with higher operational costs [196]. 

Several fusion proteins also stand out for their specific applications. Maltose-binding protein (MBP) is widely used for its ability to enhance solubility and enable purification using amylose-functionalized columns [195,197]. Thioredoxin A (TrxA) not only improves solubility but also promotes correct folding by facilitating disulfide bond formation. Green fluorescent protein (GFP) offers additional advantages, including real-time expression monitoring and functionality as a biological probe, as demonstrated in the case of GFP-equinatoxin [184,198]. The strategic combination of tags and fusion proteins is crucial for each specific case, although no universal formula exists. It is often necessary to evaluate different configurations and consider alternatives beyond conventional cellular expression systems. 

## Cell-free toxin production 

Because many toxins are difficult to express in living cells due to their inherent toxicity or complex structure cell-free protein synthesis (CFPS) has emerged as a viable alternative [184,199]. In CFPS, cell extracts enriched with transcriptional and translational machinery are supplemented with energy sources and amino acids to enable direct protein synthesis from DNA or RNA templates [200]. These systems tolerate the production of toxic proteins and allow the incorporation of non-canonical amino acids. Nevertheless, CFPS also presents limitations, such as lower yields, susceptibility of DNA/RNA to nucleases, and relatively high costs [201]. However, recent advances in automation and scalability have significantly mitigated these drawbacks, spurring growing interest from the pharmaceutical industry [184,202]. 

## Solid peptide synthesis

Solid-phase peptide synthesis (SPPS) represents another valuable alternative, particularly for small cysteine-rich toxins. This method enables the incorporation of non-natural amino acids and specific modifications, while offering precise control over the sequence [184,203]. Although generally limited to peptides of up to 50 amino acids, techniques such as segment condensation have extended its applicability to longer peptides [204,205]. While more technically demanding and less accessible than other methods, SPPS is particularly useful for toxins that are difficult to express using biological systems. The recombinant production of cnidarian toxins has facilitated not only their structural and functional analysis but also the evaluation of their pharmacological potential. These developments have paved the way for preclinical studies and biotechnological innovations based on cnidarian compounds. In the following section, we explore how these toxins are being investigated for therapeutic purposes and how their unique properties are being harnessed for drug development and biomedical applications.

## Therapeutic potential and biotechnological development of cnidarian toxins

Animal venoms represent a rich source of biologically active compounds that, when administered, can cause significant physiological damage, whether as a defense mechanism or for predation. However, these toxins also offer a promising avenue for the treatment of human diseases, as they exhibit diverse pharmacological activities. For this reason, some toxic components have been utilized in the development of novel therapeutic agents [206,207].

In this context, the U.S. Food and Drug Administration (FDA) and the European Medicines Agency (EMA) have approved several toxin-based drugs and therapies for treating various diseases. One of the earliest approved drugs in this category was captopril (approved in 1981), followed by enalapril (approved in 1985), both derived from the venom of the snake *Bothrops jararaca*. These medications function as inhibitors of angiotensin-converting enzyme (ACE) and have been widely used for hypertension treatment [207]. Another example is lixisenatide (approved in 2016 by the FDA), a synthetic drug based on a peptide identified in the venom of the Gila monster (*Heloderma suspectum*), which is prescribed for type II diabetes treatment [208]. Additionally, in 2004, the FDA and EMA approved ziconotide for the treatment of severe chronic pain. This peptide (SNX-111) is a synthetic analog of the ω-conotoxin isolated from the venom of the cone snail *Conus magus*. It blocks Cav2.2 channels, leading to the inhibition of nerve impulses and neurotransmitter release [209]. While this drug offers the advantage of being non-addictive and not inducing tolerance, its intrathecal administration significantly affects patient adherence to treatment [210].

Within this context cnidarians (e.g., sea anemones, jellyfish, corals) represent one of the least explored yet highly promising sources of bioactive toxins. These organisms produce a wide array of peptides and proteins capable of modulating ion channels, altering cell signaling, and cell lysis mechanisms of particular interest for the development of immunomodulatory, analgesic, antimicrobial, and antitumor therapeutics. A growing number of cnidarian toxins have demonstrated therapeutic potential in preclinical studies. For instance, Crassicorin-I, a neurotoxin from sea anemones, exhibits antimicrobial activity against *Bacillus subtilis*, *E. coli*, and *Salmonella enterica* [211]. Similarly, two low-molecular-mass toxins from *P. physalis* (PpV9.4 and PpV19.4) inhibit insulin secretion [212], while PpVα from *Protopalythoa variabilis* mitigates 6-hydroxydopamine-induced neurotoxicity and oxidative stress in zebrafish [213]. PcKuz3, a Kunitz-type peptide from *P. caribaeorum*, has shown promising neuroprotective properties relevant to neurodegenerative disorders [213]. Among such compounds, one of the most advanced candidates is ShK-186 (dalazatide), a recombinant analog of the ShK toxin from *S. helianthus*, which selectively blocks the Kv1.3 potassium channel, a key regulator of effector memory T cell activity. This specificity positions ShK-186 as a potential therapeutic agent for autoimmune diseases [214].

ShK-186 has demonstrated significant efficacy in preclinical models of autoimmunity, such as multiple sclerosis and pristane-induced arthritis, where a single dose administered every 2-5 days achieved comparable outcomes to daily dosing [215]. This prolonged therapeutic effect is attributed to its slow absorption at the injection site and extended residence time at the Kv1.3 channel, enabling sustained blockade for up to seven days in nonhuman primates. Based on these findings, ShK-186 progressed to phase 1 clinical trials, where it was evaluated in patients with mild to moderate plaque psoriasis [216]. The compound was well tolerated, improved skin lesions, modulated T cell activity, and reduced inflammatory mediators. Adverse events were limited to transient, mild hypoesthesia and paresthesia, with no serious complications or treatment discontinuations reported [216]. Despite these favorable outcomes, the progression to phase 2 was delayed following Kv1.3 Therapeutics LLC's decision to halt development. Nevertheless, the compound has recently re-entered the clinical pipeline under TeKV Therapeutics, which is continuing its development in the United States [217].

The therapeutic potential of cnidarian toxins has historically been constrained by challenges related to their isolation, characterization, and sustainable sourcing. These limitations include the low yield of bioactive compounds from natural specimens, small extract volumes per individual, and the high structural diversity of marine metabolites, which complicate systematic analysis. Furthermore, the ecological impact of harvesting venomous marine organisms, combined with the low concentration of target toxins and the difficulty in obtaining enough pure, target-specific samples, poses significant barriers for conventional drug discovery strategies [6,218].

In this context, heterologous expression systems have emerged as a pivotal strategy to overcome these limitations. Recombinant production enables controlled expression of cnidarian toxins in model organisms, facilitating purification, structural elucidation, and functional assays. This approach not only increases production efficiency and reproducibility but also mitigates reliance on natural sources, contributing to more sustainable research practices. The growing availability of omics data has further expanded the identification of novel bioactive peptides and proteins with therapeutic potential [165,184].

Nonetheless, despite the advances in recombinant expression, the clinical translation of cnidarian toxins still encounters significant obstacles. These include poor solubility, limited serum half-life, low oral bioavailability, restricted membrane permeability, and potential immunogenicity [219]. Additional challenges include formulation development and comprehensive safety assessments for chronic administration. Therefore, while heterologous expression offers a promising avenue for the biotechnological exploitation of cnidarian venoms, further technological and regulatory advances are required before these molecules can be established as a novel class of therapeutic agents.

## Conclusions

The study of cnidarian toxins is undergoing a profound transformation thanks to the integration of omics technologies, which have revealed the extraordinary diversity and molecular complexity of venom components. These approaches not only provide deeper insights into the gene repertoire of toxins and their evolutionary trajectories but also offer crucial information about structure - function relationships - key to elucidating their biological roles and therapeutic potential. Nevertheless, significant challenges remain in the functional validation of toxins, particularly those requiring complex folding, disulfide bonds, or post-translational modifications, all of which complicate their recombinant expression in heterologous systems. In this context, advances in synthetic biology and cell-free protein synthesis offer promising alternatives to overcome these limitations; however, it is important to note that they do not yet provide the scalability required for industrial production. This raises a key question: How might peptide synthesis technologies and cell-free systems be integrated to accelerate the characterization of new toxins?

The therapeutic and biotechnological potential of cnidarian toxins - ranging from ion channel modulators to antitumor agents - positions them as an underexplored source of bioactive compounds. However, the realistic translational application of these molecules will depend on our ability to produce enough biologically active toxins, understand their mechanisms of action at the molecular level, and minimize off-target effects.

Artificial intelligence (AI) emerges as a strategic tool to accelerate large-scale functional analysis of toxins. For example, machine learning models trained to predict biological functions, structural properties, and molecular targets from omics data can reduce the reliance on time-consuming and expensive experimental approaches. Moreover, AI can assist in establishing prioritization systems to select toxins with the greatest therapeutic potential, optimize sequences for recombinant expression, and even design synthetic variants with improved properties.

As the field progresses, several key questions remain: What are the ecological and evolutionary drivers of toxin diversity in cnidarians? How can we streamline the process from gene discovery to functional characterization and therapeutic development? What are the major regulatory and technical barriers preventing cnidarian toxins from reaching clinical trials? What criteria should be established to prioritize therapeutic candidates among the hundreds of toxins identified through omics approaches? How might artificial intelligence improve the prediction and prioritization of toxin function? What role will interdisciplinary collaborations - among bioinformatics, pharmacology, and marine biology - play in the future of cnidarian toxin research?

An integrative and interdisciplinary approach - combining omics sciences, structural biology, artificial intelligence tools, synthetic expression systems, and pharmacological testing - will be essential to unlock the full potential of cnidarian venoms. This review underscores the need to continue innovating and strengthening collaboration in this emerging field, which not only promises novel therapeutic strategies, but also a deeper understanding of venom biology.

### Abbreviations

AMPs: antimicrobial peptides; ASICs: acid-sensing ion channels; CDPs: cysteine-dense peptides; CFPS: cell-free protein synthesis; cPLA2: cytosolic phospholipase A2; EMA: European Medicines Agency; FDA: Food and Drug Administration; fMSI: functional mass spectrometry imaging; GFP: green fluorescent protein; HA: hyaluronic acid; IMAC: immobilized metal affinity chromatography; KTPIs: Kunitz-type peptidase inhibitors; KTx: neurotoxin that interacts with Kv channels; Kv: voltage-gated potassium; MSI: mass spectrometry imaging; nAChRs: nicotinic acetylcholine receptors; NaTx: neurotoxin that interacts with Nav channels; Nav: voltage-gated sodium; PFTs: pore-forming toxins; PLA2: phospholipase A2; PLB: phospholipase B; PTM: post-translational modifications; SCRiPs: small cysteine-rich peptides; sPLA2: secretory phospholipase A2; SPPS: solid-phase peptide synthesis; 

ST: spatial transcriptomics; TRPA1: transient receptor potential ankyrin 1; TRPV1: transient receptor potential vanilloid 1.

## Availability of data and materials

 No new datasets were generated or analyzed in this review article. 
